# Identification of preoperative predictors for acute postsurgical pain and for pain at three months after surgery: a prospective observational study

**DOI:** 10.1038/s41598-021-95963-y

**Published:** 2021-08-12

**Authors:** Regina L. M. van Boekel, Ewald M. Bronkhorst, Lilian Vloet, Monique A. M. Steegers, Kris C. P. Vissers

**Affiliations:** 1 Department of Anesthesiology, Pain and Palliative Medicine, Radboud University Medical Center, intern 714, PO Box 9101, 6500 HB Nijmegen, The Netherlands; 2 Department for Health Evidence, Radboud University Medical Center, Nijmegen, The Netherlands; 3 Research Department of Emergency and Critical Care, HAN University of Applied Sciences, Nijmegen, the Netherlands; 4 Institute for Health Sciences IQ Healthcare, Radboud University Medical Center, Radboud Nijmegen, The Netherlands; 5 Department of Anesthesiology, Amsterdam University Medical Center Location VU, Amsterdam, The Netherlands

**Keywords:** Health care, Medical research, Risk factors

## Abstract

Identifying patients at risk is the start of adequate perioperative pain management. We aimed to identify preoperative predictors for acute postsurgical pain (APSP) and for pain at 3 months after surgery to develop prediction models. In a prospective observational study, we collected preoperative predictors and the movement-evoked numerical rating scale (NRS-MEP) of postoperative pain at day 1, 2, 3, 7, week 1, 6 and 3 months after surgery from patients with a range of surgical procedures. Regression analyses of data of 2258 surgical in- and outpatients showed that independent predictors for APSP using the mean NRS-MEP over the first three days after surgery were hospital admittance, female sex, higher preoperative pain, younger age, pain catastrophizing, anxiety, higher score on functional disability, highest categories of expected pain, medical specialty, unknown wound size, and wound size > 10 cm compared to wound size ≤ 10 cm (RMSE = 2.11). For pain at three months, the only predictors were preoperative pain and a higher score on functional disability (RMSE = 1.69). Adding pain trajectories improved the prediction of pain at three months (RMSE = 1.37). Our clinically applicable prediction models can be used preoperatively to identify patients at risk, as well as in the direct postoperative period.

## Introduction

Surgical procedures commonly cause acute postsurgical pain (APSP), with a clinical prevalence as high as 80%^1^. APSP treatment should provide a better quality of life and comfort for patients, minimize postsurgical complications including chronic postsurgical pain^2^, and improve general outcome^3,4^ while avoiding higher healthcare costs and low patient dissatisfaction. Current (multimodal) strategies to prevent pain seem insufficient, mostly due to a lack of consistent organizational aspects and a lack of focus on organized approaches to identify and assure procedure and patient-specific pain management^1,5,6^. There is evidence that improving APSP strongly depends on preventing, timely identifying, and quantifying postoperative pain. Several (bio)markers have been identified and validated as predictors for APSP^7,8^. Identifying early postoperative pain may prevent pain from becoming chronic^9^. A recent study showed that several potential predictors for APSP demonstrated strong correlation with predictors for chronic postsurgical pain (CPSP), such as age, sex, obesity and emotional state^10^. Other studies identified predictors for CPSP also used in APSP prediction models, such as preoperative numerical rating scale (NRS) score^11^ and catastrophizing^12^. Furthermore, there is evidence that problematic pain resolution may be predicted by preoperative pain, early postoperative pain, and progressive evolving pain observed during long term pain trajectories^13,14^.

## Objectives

We aimed to identify preoperative predictors for acute postsurgical pain and for pain at three months after surgery. Using preoperative predictors and pain trajectories, we will develop applicable clinical prediction models for APSP as well as for pain at three months after surgery. We hypothesize that using the combination of preoperative predictors and pain trajectories we will be able to better predict pain at three months after surgery than when using preoperative predictors alone.

## Methods

### Study design

For this prospective observational cohort study, we conducted a prospective patient questionnaire at the Radboud University Medical Center. The Institutional Review Board of the Radboud University Medical Center (Nijmegen, the Netherlands) ethically approved the study (authorization number: 2012/430). All methods were performed in accordance with the relevant guidelines and regulations applicable to research with human subjects, such as the World Medical Association Declaration of Helsinki and the Medical research Involving Human Subjects Act. Written informed consent was obtained from all participants. All participating patients received the usual care customary for each of the surgical procedures and specialties, according to the standardized hospital surgical pain protocol (see Supplementary Information table S1) Preliminary data was analyzed and published in an earlier study about the relationship between APSP and complications after surgery^2^.

### Participants

All patients who underwent scheduled surgery between November 2012 and June 2018 were considered eligible for participation. Although all patients of all surgical specialties were considered eligible, exclusion criteria were: (1) younger than 18 years of age (2) procedures outside the operation room (3) emergency surgery and (4) hospitalization in intensive care units. All patients were recruited after their visit to the anesthesia preoperative evaluation clinic of the Department of Anesthesiology. Once every two weeks, medical research assistants sequentially contacted these patients by telephone. They asked them to participate and to return a questionnaire including written informed consent. Patients who agreed and returned the questionnaire were enrolled in the study. They were asked to fill in questionnaires at seven time-points, one preoperatively (time-point 1) and six postoperatively (time points 2–7), i.e. day one, day two, day three, day seven, week 6, and month three.

### Identification of potential predictors for APSP

On the day before surgery, each participant received a journal to record potential predictor variables including their age, sex, weight, height, surgical procedure, hospital admittance (yes/no), expected wound size (> 10 cm, < 10 cm, or unknown), and pain intensity (both at rest and during normal movement)^8^. Other variables measured were: category of expected pain, medical specialty, preoperative pain intensity, functional disability, anxiety, need for information, catastrophizing, and pain sensitivity.

Pain intensity was measured with an 11-point numerical rating scale (NRS) with end points representing the extremes of the pain experience: 0 = “no pain at all” and 10 = “worst possible pain”^8^.

We used the Pain Disability Index Dutch language version (PDI) to measure functional disabilities.The PDI is a widely used and studied instrument for measuring and evaluating disability associated with pain^15,16^. The PDI is a 7-item questionnaire to investigate the magnitude of the self-reported disability in different situations such as work, leisure time, daily life activities, and sports. The questionnaire is constructed on an 11-item numeric rating scale in which 0 means no disability and 10 maximum disability.

The Amsterdam Preoperative Anxiety and Information scale (APAIS) was used to provide information about anxiety and need for information^17^. The APAIS consists of six questions, each scored on a 5-point Likert scale from 1 (not at all) to 5 (extremely). The APAIS is specifically designed to assess the patient's preoperative anxiety score (4 questions, score range 4–20) and an information-seeking behavior score to assess the patient's need for information regarding the scheduled surgery and anesthesia (2 questions, score range, 2–10)^8^.

We used the Pain Catastrophizing Scale (PCS), a self-evaluating questionnaire to measure catastrophizing, generally described as an absurd negative orientation towards hurtful stimuli and of great importance in coping with pain^18^. The PCS consists of 13 questions. People are asked to indicate the degree to which they have thoughts and feelings when they are experiencing pain using the 0 (not at all) to 4 (all the time) scale. A total score is yielded (ranging from 0–52).

The Pain Sensitivity Questionnaire (PSQ) was used to measure preoperative pain sensitivity^19,20^. The PSQ consists of 17 questions which describe situations in daily life; respondents score their pain intensity for these situations on an NRS by scoring 0 (not painful) to 10 (strongest pain imaginable).

Surgery-related information was operationalized by using medical specialty, type of surgery, and wound size. As there are a large number of surgical procedures, we used the model developed by Janssen et al. to classify them in categories of expected postoperative pain^8^. They identified 27 groups of surgical procedures based on clinical experience, current practice, and interviews with surgeons and anesthesiologists, taking into account the univariate association between each surgical group and early severe acute postoperative pain. It shows five classes of surgical procedures ordered by increasing incidence of severe acute postoperative pain (NRS ≥ 6) occurring within the first hour after surgery, displaying 5 categories, from ‘lowest’ to ‘highest’ level of expected pain. For our study, we used these five categories of expected pain, hereafter referred to as the Janssen classification of expected pain. Furthermore we asked for expected wound size. Additionally, we asked for the actual wound size on the first day after surgery.

### Outcome measures

Pain intensity has been used to study pain and to develop a prediction model for acute and chronic postoperative pain, so as outcome measure, we collected the movement-evoked pain intensity at the same postoperative time-points mentioned above^3,8,12,21^.

Postoperatively, the journal was used to enter the results of the assessment of pain. Patients were asked to rate their pain with the NRS, both at rest and movement-evoked, in the morning after bathing and getting dressed on each of the six post-operative time points^22–24^. Since movement-evoked pain (MEP) has a negative effect on recovery after surgery^25^, NRS-MEP was used in this study as dependent variable in the prediction models, and not as a pain score measuring pain at rest.

### Prediction rule for pain at three months after surgery

Recent studies show that several potential APSP predictors are also predictors for CPSP, such as age, sex, obesity, emotional state^10^, preoperative NRS score^11^ and catastrophizing^12^.There is also evidence that problematic pain resolution may be predicted by preoperative pain and early postoperative pain, as well as pain trajectories^13,14^. Therefore we defined the NRS-MEP at three months after surgery as an outcome to be able to investigate a prediction model for pain at three months after surgery, using the predictors for APSP and pain trajectories. The pain trajectory, normally resolving in intensity over days, is a longitudinal characterization of acute pain as a growth curve. The psychometric goal of growth curve modeling is to estimate the true, dynamic course of acute pain resolution in each individual. A basic assumption of this approach is that acute pain is an attribute of the individual patient that follows a dynamic trajectory, with individuals differing in the specific features of their unique pain trajectories^26^.

### Statistical analysis

The incidence of postoperative pain, presented as moderate pain (NRS-MEP 4–7) and severe pain (NRS-MEP 8–10) were calculated. PCS, PDI and PSQ questionnaires with one or two missing sub-questions were explicitly retained in the analysis to limit the amount of excluded data and to enhance generalizability^27,28^. For the other variables, only complete cases were retained^29^.

To quantify the prediction model for APSP, we estimated four linear regression models. In the first three models, the dependent continuous variable was NRS-MEP, calculated per postoperative day: day one, day two and day three. In the last model, the dependent continuous variable was the NRS-MEP, calculated as the mean movement evoked pain intensity over all three first days after surgery. All values of NRS-MEP were used without a predefined cut-off score, thereby increasing the clinical relevance of our prediction model^21,30,31^. The predictors were all the items derived from the patient questionnaires and the surgery-related information. We used a least absolute shrinkage and selection operator (Lasso) regression. Shrinkage is where data values are shrunk towards a central point, like the mean. Lasso is a regression analysis method that performs both variable selection and regularization in order to enhance the prediction accuracy and interpretability of the statistical model it produces. We chose the Lasso procedure because it performs L1 regularization, which adds a penalty equal to the absolute value of the magnitude of coefficients. This type of regularization can result in sparse models with few coefficients; some coefficients can become zero and thus be eliminated from the model. Larger penalties result in coefficient values closer to zero, which is the ideal for producing simpler models. For the regularization parameter λ, we chose the λ  =  λ 1SE which can be interpreted as valuing reduction of model complexity over model precision. We randomly used 80% of the data as learning set and 20% as test set to ensure selection bias. To test the accuracy of our models, we used the Root Mean Square Error (RMSE), i.e. the standard deviation of the residuals (prediction errors). The smaller the RMSE, the better the fit.

Furthermore, we analyzed whether or not pain trajectories were able to predict pain at three months after surgery. The individual trajectory calculated across six measures (using the order data points) may be a linear fit, and each patient’s trajectory has two key features: (1) the intercept, or initial pain level; and (2) the slope, or rate of pain resolution. As the time intervals between pain measurements were chosen to be larger as time passed, the trajectories were at risk of being heavily influenced by the final point in time. Therefore, the time points at which time was measured were coded in equidistant time points in the trajectories analysis.

Lastly, we analyzed whether adding the APSP predictors to the pain trajectory model would lead to a better model to predict pain at three months after surgery. We did this both for the short-term pain trajectory, the first three days, as well as for the longer term, including the first week. Using this approach both for the short and longer-term trajectories for each patient, an intercept and slope was acquired. Subsequently, prediction with Lasso regression was repeated twice, by adding either the information for the short-term or longer-term trajectory to the predictors. Adding the pain trajectory of six weeks after surgery was not considered clinically relevant.

Data were analyzed using SPSS (IBM Corp. Released 2013. IBM SPSS Statistics for Windows, Version 22.0. IBM Corporation, Armonk, NY, USA) and the open-source statistical analysis software R (R version 3.6.2; The R Foundation for Statistical Computing, Vienna, Austria; the glmnet package was used for Lasso regression. In all analyses a P-value < 0.05 was considered significant.

## Results

### Patient characteristics

**Figure 1﻿:**
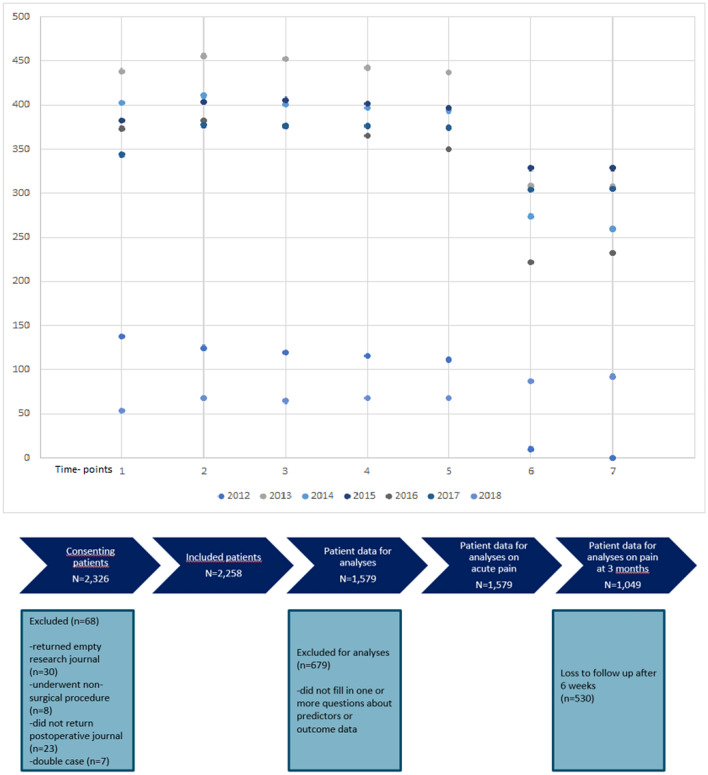
Cohort timeline. For each study year, the frequency of included patients per time-point of the study is shown in addition to the flowchart of patient recruitment and inclusion.

Nearly 6000 patients were asked to participate in the study (5,923). Response rate was 39.2%. We used the data of 2,258 surgical in- and outpatients for the analyses. As we only worked with complete cases, 679 cases were excluded for missing predictor variables or outcome data. Further reasons for exclusion can be found in Fig. 1. (see Fig. 1). Table 1 summarizes the demographic information of our cohort, showing that 48% of our patients in the dataset were male. (see Table 1) More than two thirds of the cohort were inpatients. Of the total cohort, more than two thirds of the patients had a preoperative movement-evoked NRS 0–3 (67%), 21% had an NRS 4–7, and 12% had an NRS 8–10. Of the patients with a preoperative NRS 8–10, 11% had an NRS 8–10 at three months after surgery. No significant differences were found between the total cohort, the cohort used for the acute pain analyses, and the cohort used for the analyses for pain at three months.

**Table 1:** Patient characteristics of the study population

	Total	Acute pain	Pain at 3 months
	(n = 2,258)	(n = 1,579)	(n = 1,049)
**Hospital admittance (n, %)**			
yes	1,566 (69.4)	1,075 (68.1)	713 (68.0)
no	692 (30.6)	504 (31.9)	336 (32.0)
**Sex (n, %)**			
male	1,083 (48.0)	764 (48.4)	513 (48.9)
female	1,175 (52.0)	815 (51.6)	536 (51.1)
Preop movement evoked pain (median, IQR)	1.0 (0.0–5.0)	1.0 (0.0–5.0)	1.0 (0.0–4.0)
Preop pain acceptable	1,828 (81.0)	1,334 (84.5)	901 (85.9)
Age	58.8 (47.4–67.1)	57.7 (46.6–66.5)	59.3 (48.7–66.7)
BMI	25.6 (23.3–28.4)	25.5 (23.3–28.3)	25.5 (23.1–28.1)
PCS total	11.0 (4.0–21.0)	10.0 (3.0–19.0)	9.8 (3.0–18.0)
PSQ total mean	44.0 (30.0–61.0)	43.0 (29.0–59.0)	45.5 (29.5–58.0)
APAIS anxiety	3.0 (1.0–6.0)	3.0 (1.0–6.0)	3.0 (1.0–6.0)
APAIS information	3.0 (1.0–5.0)	3.0 (1.0–5.0)	3.0 (2.0–5.0)
PDI total	10.0 (0.0–31.0)	8.0 (0.0–27.0)	7.0 (0.0–25.0)
**Category expected pain**			
Category expected pain 1	246 (10.9)	165 (10.4)	107 (10.2)
Category expected pain 2	377 (16.7)	271 (17.2)	172 (16.4)
Category expected pain 3	504 (22.3)	357 (22.6)	242 (23.1)
Category expected pain 4	650 (28.8)	454 (28.8)	304 (29.0)
Category expected pain 5	481 (21.3)	332 (21.0)	224 (21.4)
**Medical specialty (n,%)**			
General surgery	451 (20.0)	319 (20.2)	211 (20.1)
Orthopedics	364 (16.1)	260 (16.5)	175 (16.7)
Urology	391 (17.3)	263 (16.7)	175 (16.7)
Gynecology	222 (9.8)	161 (10.2)	95 (9.1)
ENT	238 (10.5)	178 (11.3)	124 (11.8)
Eye surgery	63 (2.8)	48 (3.0)	34 (3.2)
Plastic surgery	197 (8.7)	132 (8.4)	87 (8.3)
Neurosurgery	141 (6.2)	91 (5.8)	62 (5.9)
Oro-maxillofacial surgery	95 (4.2)	59 (3.7)	40 (3.8)
Other surgeries	96 (4.3)	68 (4.3)	46 (4.4)
**Expected wound size (n,%)**			
Expected wound size < 10 cm	965 (44.7)	724 (45.9)	465 (44.3)
Expected wound size > 10 cm	630 (27.9)	465 (29.4)	322 (30.7)
Expected wound size unknown	565 (25.0)	390 (24.7)	262 (25.0)
**Real wound size (n,%)**			
Wound size < 10 cm	971 (43.0)	730 (46.2)	488 (46.5)
Wound size > 10 cm	825 (36.5)	615 (38.9)	407 (38.8)
Wound unknown	363 (16.1)	234 (14.8)	154 (14.7)

*BMI* Body mass index, *PCS* Pain Catastrophizing Scale, *PSQ* Pain Sensitivity Questionnaire, *APAIS* Amsterdam Pain Anxiety and Information Scale, *PDI* Pain Disability Index, *tot* means total score; *ENT* Ear Nose Throat surgery.

### Incidence of postoperative pain

The incidence of postoperative pain measured at the different time points is shown in Table 2. (see Table 2). On day 1 after surgery, 55% of the patients reported an NRS-MEP between 4 and 10, of which 15% reported an NRS between 8 and 10. In the first three days, 54% reported a mean NRS-MEP 0–3, 40% 4–7, and 7% 8–10. At 3 months after surgery, 12% of patients reported an NRS-MEP 4–10, of which 2% had an NRS-MEP 8–10.

**Table 2:** Incidence of movement-evoked pain intensity measured by numerical rating scale at the different time-points of the study.

	Movement-evoked numerical rating scale (NRS-MEP)		
	0–3 (%)	4–7 (%)	8–10 (%)
Preoperative	67	21	12
Day 1	45	40	15
Day 2	53	36	11
Day 3	61	32	7
Week 1	72	24	4
Week 6	84	13	3
Month 3	88	10	2

### Prediction model acute postsurgical pain

Table 3 shows the prediction model for the mean NRS-MEP for each of the first three postoperative days, as well as the prediction model for the first three days combined. (see Table 3). Meaningful predictors in one of the four models were hospital admittance, female sex, higher preoperative movement evoked pain, younger age, higher score on catastrophizing (PCS total score), higher APAIS anxiety score, higher score on functional disability (PDI total score), higher Janssen classification of expected pain, medical specialty, unknown wound size, and wound size > 10 cm compared to wound size ≤ 10 cm. The prediction model for the mean NRS-MEP over the first three days combined contained the most predictors and showed the lowest RMSE of 2.11. Acceptable preoperative pain, APAIS information, PSQ total score, BMI and expected wound size did not seem to be of value in the final models.

**Table 3:** Estimates obtained from least absolute shrinkage and selection operator (Lasso) regression analyses showing the prediction models of acute postsurgical pain. (N = 1,579) Independent variables are the preoperative predictors. The dependent variables are movement-evoked acute postsurgical pain (NRS-MEP) at day 1, 2, 3 and over the first three days after surgery.

Preoperative predictors	Day 1			Day 2			Day 3			Day 123		
	Est	RMSE	R^2^	Est	RMSE	R^2^	Est	RMSE	R^2^	Est	RMSE	R^2^
		2.52	0.162		2.44	0.163		2.18	0.176		2.11	0.207
Intercept	4.082			3.564			3.099			3.669		
**Hospital admittance**	0.018						0.087			0.091		
**Female sex**	0.101									0.019		
**Preoperative movement evoked pain**	0.148			0.130			0.148			0.146		
**Age**	− 0.017			− 0.016			− 0.017			− 0.021		
**PCS total**	0.010			0.019			0.014			0.015		
**APAIS anxiety**	0.051			0.028			0.041			0.047		
**PDI total**	0.012			0.011			0.008			0.012		
**Category expected pain**												
Category expected pain 1												
Category expected pain 2												
Category expected pain 3												
**Category expected pain 4**	0.094											
**Category expected pain 5**	0.138			0.286			0.161			0.272		
**Medical specialty**												
General surgery												
Orthopedics												
**Urology**										0.053		
Gynecology												
**ENT**	− 0.157									− 0.087		
Eye surgery												
Plastic surgery												
Neurosurgery												
Oro-maxillofacial surgery												
Other surgeries												
Wound size unknown*	0.187											
Wound size > 10 cm*				0.197			0.170			0.265		

Est means estimate; RMSE means Root Mean Square Error; R^2^ means R-squared; PCS means Pain Catastrophizing Scale; APAIS means Amsterdam Pain Anxiety and Information Scale; PDI means Pain Disability Index. Meaningful predictors in one of the four prediction models are displayed in bold font.

* compared to wound size ≤ 10 cm.

Table 4 shows the results of the analyses performed to predict pain at three months after surgery using pain trajectories. (see Table 4). For example: the equation of the prediction model of postsurgical pain at three months after surgery using the pain trajectory of NRS-MEP on day 1,2,3 is:

$${\text{PostsurgicalPain}}_{{{\text{3M}}}} = 0.{125} + 0.{\text{325 Intercept}}_{{{\text{NRS}} - {\text{MEP}} - {123}}} + 0.{\text{836 Slope}}_{{{\text{NRS}} - {\text{MEP}} - {123}}} + {\text{residual}}$$

**Table 4:** Estimates obtained from least absolute shrinkage and selection operator (Lasso) regression analyses showing the prediction model of postsurgical pain at three months after surgery. (N = 1,049).

	Intercept	Individual intercept	(CI)	P-value	Slope	(CI)	P value	R^2^	RMSE
NRS-MEP on day 1,2,3	0.125	0.325	0.285–0.365	<0.001	0.836	0.710–0.961	<0.001	0.142	1.85
NRS-MEP on day 1,2,3,7	0.086	0.413	0.371–0.455	<0.001	1.476	1.304–1.647	<0.001	0.198	1.79
NRS-MEP on day 1,2,3,7, week 6	0.034	0.669	0.631–0.708	<0.001	3.364	3.178–3.551	<0.001	0.460	1.47

Independent variables are pain trajectories over different time points. The dependent variable is movement-evoked pain (NRS-MEP) at three months after surgery.

*NRS-MEP* movement-evoked numerical rating scale, *CI* confidence interval, R^2^ means R-squared; RMSE means Root Mean Square Error.

In this equation the Intercept_NRS-MEP-123_ for each patient is the intercept of the patient’s regression line for the NRS-MEP at day 1, 2 and 3. The Slope_NRS-MEP-123_ is the slope of the same individual regression. In the other two equations, the intercept and slope are calculated per patient as described above, but using the NRS-MEP for day 7 and week 6 respectively, in the individual regressions.

Pain trajectories alone were able to significantly (P < 0.001) predict postsurgical pain at three months with an RMSE of 1.85; 1.79; 1.47 for NRS-MEP on day 1,2,3; day 1,2,3,7; day 1,2,3,7,week 6, respectively. The more data points used, the better the prediction and the smaller the RMSE.

In the prediction model for pain at three months without the pain trajectories, only higher preoperative movement evoked pain and a higher score on functional disability (PDI total score) were relevant. (see Table 5).

**Table 5:** Estimates obtained from least absolute shrinkage and selection operator (Lasso) regression analyses showing the prediction model of postsurgical pain at three months after surgery. (N = 1,049).

	Without pain trajectory			With pain trajectory					
				slope day 1,2,3			slope day 1,2,3,7		
**Preoperative predictors**	**est**	**RMSE**	**R**^**2**^	**est**	**RMSE**	**R**^**2**^	**est**	**RMSE**	**R**^**2**^
		1.69	0.243		1.46	0.427		1.37	0.501
Intercept	0.659			-1.673			− 1.508		
Individual intercept				0.261			0.425		
Slope				0.734			1.712		
**Preoperative predictors**				1.959			1.668		
**Preoperative movement evoked pain**	**0.150**								
**PDI total**	**0.005**								

Independent variables are preoperative predictors with and without pain trajectories over different time points. The dependent variable is movement-evoked pain (NRS-MEP) at three months after surgery.

*RMSE* Root Mean Square Error, *PDI* Pain Disability Index, *R*^*2*^ R-squared.

Finally, we analyzed whether adding the preoperative predictors to the pain trajectory model led to a better model for predicting pain at three months after surgery. Table 5 shows the prediction model without the pain trajectories (RMSE = 1.69), as well as the prediction model with the added pain trajectories. For the latter, two models are displayed: one using the data of the first three days after surgery (RMSE = 1.46) and one using the data of the first seven days after surgery (RMSE = 1.37).

The first equation of the prediction model of postsurgical pain at three months after surgery using only preoperative predictors, without pain trajectory, is:

$${\text{PostsurgicalPain}}_{{{\text{3M}}}} = 0.{659} + 0.{15}0{\text{ PreoperativeMovementEvokedPain}} + 0.00{\text{5 PDI}}_{{{\text{total}}}} + {\text{residual}}$$

The second equation, with the pain trajectory for the first three days included is:

$${\text{PostsurgicalPain}}_{{{\text{3M}}}} = - {1}.{673} + 0.{\text{261 Intercept}}_{{{\text{NRS}} - {\text{MEP}} - {123}}} + 0.{\text{734 Slope}}_{{{\text{NRS}} - {\text{MEP}} - {123}}} + {1}.{\text{959 PreoperativePredictor}} + {\text{residual}}$$

In this equation, the PreoperativePredictor is the prediction in the model using preoperative predictors only, so that is 0.150 PreoperativeMovementEvokedPain + 0.005 PDI_total._ In the third equation, the intercept and slope of the first seven days after surgery was included.

The prediction model using the data of the preoperative predictors and the data of the first week after surgery results in the best model with the lowest RMSE.

## Discussion

We developed clinical applicable prediction models for APSP using the NRS-MEP as outcome measure per postoperative day (days, 1, 2 and 3) and as the mean NRS-MEP over all three days after surgery combined. Meaningful predictors in one of the four models for APSP were hospital admittance, female sex, higher preoperative movement evoked pain, younger age, higher score on catastrophizing (PCS total score), higher APAIS anxiety score, higher score on functional disability (PDI total score), higher Janssen classification of expected pain, medical specialty, unknown wound size, and wound size > 10 cm compared to wound size ≤ 10 cm. We also developed prediction models for postsurgical pain at three months using the NRS-MEP at three months after surgery as outcome measure, with the preoperative predictors, pain trajectories, and a combination of these. For pain at three months, only higher preoperative movement evoked pain and a higher score on functional disability (PDI total score) were relevant. As hypothesized, the prediction model using the data of the preoperative predictors and the pain trajectories of the first week after surgery gives the best fit.

We found that moderate and severe pain occurred in 55% of the patients on the first day after surgery, 47% on the second and 39% on the third. In 2008, 41% of 1,490 inpatients reported moderate or severe pain on day 0, and 30%, 19%, 16% and 14% on day 1, 2, 3 and 4, respectively^32^. The prevalence of acute postsurgical pain in our study is comparable with other studies, although figures vary because of methodological differences, such as the definition of moderate or severe pain and pain assessment at rest or movement-evoked^33^.

We noted that of the patients with a preoperative NRS 8–10 (12%), 11% of these patients had an NRS 8–10 at three months after surgery. Furthermore, prospective studies showed that the long duration of preoperative chronic pain and more severe intensity of this pain are associated with a higher likelihood of reporting pain persistence after surgery^34^. In our non-selected surgical population with patients of all types of surgery, this may imply that a certain number of patients will continue to have a high pain score unless medically treated. This phenomenon was not recognized in other studies investigating patients undergoing homogeneous surgical procedures. For example, Montes and co-workers demonstrated that around 20% of their included patients (inguinal hernia repair, hysterectomy and thoracotomy) had preoperative pain located in the surgical area or in other areas^10^. The authors showed in their supplemental information that approximately 27% of the patients developing chronic postsurgical pain had preoperative pain. Nevertheless, this observation is interesting and will need further study.

Several predictive markers have been described in other studies^8,10,11,35,36^. In accordance with these studies, we found a significant independent predictive value for sex, age^36,37^, preoperative pain, wound size, the Janssen classification of expected pain, hospital admittance^38^, as well as for PCS^35^. BMI was non-significant in our study, which is in line with several other studies. In a study looking at patients after a total hip arthroplasty, increased BMI was not associated with increased pain or analgesic consumption in the first 24 h after surgery^39^. This lack of association was also shown in pain following laparoscopic gastric bypass surgery^40^ and ankle fracture surgery^41^. However, in an earlier study BMI was found to be a predictor for poor pain control^42^. Only the APAIS anxiety was relevant in our study, in contrast to Janssen et al. We also found APAIS information to be relevant^7^ which may be inherent to the use of our Lasso procedure, performing L1 regularization, which results in sparse models with few coefficients, thus producing a simple model. Medical specialty was not found to be important in our study. In other studies with patients undergoing only one type of surgery, apparently this is no issue^40,43^. But in a larger study of more than 50,000 patients, medical specialty was not considered important, however duration of surgery was found to be relevant^44^. This may suggest that not the type of surgery is important, but its duration.

Pain sensitivity measured by PSQ total score was not significantly relevant in our model. This contrasts with Rehberg et al. showing that the PSQ could be used as an important predictor to identify patients at risk for acute postoperative pain following breast cancer surgery^43^. In a prediction model, highly correlated variables may explain much the same variability in the outcome. We used the Lasso method resulting in sparse models with few coefficients, which selected only the best one of the correlated variables. We included PCS in our study PCS. Earlier studies have mentioned a relationship between pain catastrophizing and pain sensitivity, however these mechanisms are not yet clear^45,46^. Therefore, PSQ and PCS were highly correlated, and PCS came out as best in our models. This may have a positive influence on clinical applicability: the PSQ consists of 17 questions and can be prone to loss of information, thus reducing the simplicity and applicability of our preoperative model.

We identified predictors for early and late (3 months) postsurgical pain. Ideally the prediction model for APSP will be used before surgery to identify patients at risk, so that preventive interventions can be scheduled. We not only developed a prediction model to initiate preventive measures before surgery in patients at greater risk, but we also made a prediction model to be used in the first days after surgery. This may be important, as poor acute pain management is known to be a warning for potential persistent pain, and therefore it should always be taken into account^33^. The importance of identifying patients at risk before and during admittance has been described in studies on transitional pain services^9,47^. As mentioned by Katz et al., an acute pain service can play a role in referring for further treatment after discharge. This is especially advisable if patients have intense or prolonged postsurgical pain, high opioid use, notable emotional distress, or if there is a need for ongoing expert pain management consultation and care. The need for patient-specific pain interventions based on risk profile can be supported by our prediction model both preoperatively as well as in the early postoperative phase, using the preoperative predictors as well as the pain trajectories.

An important strength of our study is the prospective recruitment of a large consecutive cohort of patients, far exceeding the rule of thumb of having at least 100 events per predictor^48^. Another strength is the broad definition of postoperative pain using all values of the NRS which increases the clinical relevance of our prediction model^21^. Our model can also be updated specific to each procedure, so patients will benefit even more from the risk identification and preventive measures taken. Our cohort was highly heterogeneous, with a broad spectrum of operations, and included inpatients and outpatients from different specialties thereby inducing bias, however we deliberately chose for this methodology. A smaller cohort, with a limited number of procedures, would have made the analysis more homogenous but would have limited the broader clinical perspective. By using the regular standards of care, we maximized the clinical applicability.

A critical note in this regard was the high rate of missing data. In prediction studies, missing data should be as limited as possible^28^. We decided however not to use imputing strategies. Without imputing data, we provided results that best represent the overall population. Furthermore, we lost 530 patients that did not return the questionnaire after three months (loss to follow-up of 34%). However, our analysis of the patient characteristics of the study population for APSP and the study population of pain at three months revealed no important differences. Another limitation is the definition of pain at three months. We only used the patient-reported movement-evoked pain at three months after surgery. We did not include further questions about the other parts of the definition of chronic postsurgical pain, i.e. pain after surgery localized to the surgical field that lasts for at least three months, and cannot be better explained by another cause^49^. Although the patient information about the study implied this, we cannot be sure that the pain at three months is chronic postsurgical pain. Therefore, we used the term “pain at three months after surgery”.

## Conclusions

In this prospective cohort study, we developed a clinically applicable prediction model for acute postsurgical pain and for pain at three months after surgery, using predictors and pain trajectories. The prediction model for the mean NRS-MEP for the combined first three postoperative days contained the most predictors and was most accurate for predicting acute postsurgical pain. The prediction model using the data of the preoperative predictors and the data of the first week after surgery is the best model for predicting pain at three months after surgery. Our models can be used to identify patients at risk preoperatively, as well as in the direct postoperative period. Further studies need to focus on clinical application of the models.

## Supplementary Information

Supplementary Information.
