# Supplementary material for: Identification of preoperative predictors for acute postsurgical pain and for pain at three months after surgery: a prospective observational study
Source: Sci Rep. 2021 Aug 12;11:16459. doi: 10.1038/s41598-021-95963-y (PMC8361098; doi:10.1038/s41598-021-95963-y)
Supplement: Supplementary file 1 — Supplementary Information. [file 41598_2021_95963_MOESM1_ESM.docx]

| **Supplementary information 1** |  |
| --- | --- |
| **Table S1: Standardized protocol for postoperative pain treatment technique and medication used.** | |
| **Pain treatment technique** | **Medication** |
| Acetaminophen | 4 dd 1000 mg daily |
| Diclofenac | 3 dd 50 mg daily |
| Patient controlled intravenous analgesia (PCIA) | Morphine 2 mg ml^-1^, bolus 1 mg, lock-out time 6 minutes |
|  | Piritramide 2 mg ml^-1^, bolus 1 mg, lock-out time 6 minutes |
|  | Fentanyl 50 μg ml^-1^, bolus 25 µg, lock-out time 6 minutes |
| Continuous epidural analgesia (CEA) | bupivacaine 0.75% mixed with morphine 0.2 mg ml^-1^ 1-2 ml hour^-1^ |
|  | ropivacaine 0.2% 4-12 ml hour^-1^ |
|  | ropivacaine 0.2% mixed with sufentanil 1 µg ml^-1^ 4-12 ml hour^-1^ |
| Patient controlled epidural analgesia (PCEA) | ropivacaine 0.2% mixed with sufentanil 1 µg ml^-1^ 4-10 ml hour, bolus 2 mg, lock-out time 20 minutes |
| Continuous peripheral nerve blocks (CPNB) | ropivacaine 0.2% 5-10 ml hour^-1^ |
| Esketamine | 5-10 mg hour^-1^ |
